# cGAS-STING signaling: a therapeutic target in inflammatory bowel disease and related colorectal cancer

**DOI:** 10.3389/fimmu.2025.1709908

**Published:** 2026-01-15

**Authors:** Xi Chen, Xiaohua Tang, Sixuan Chen, Yang Ye, Fei Mao

**Affiliations:** 1 Department of laboratory Medicine, School of Medicine, Jiangsu University, Zhenjiang, Jiangsu, China; 2 Department of Laboratory Medicine, the Affiliated People's Hospital, Jiangsu University, Zhenjiang, Jiangsu, China; 3 The People’s Hospital of Danyang, Affiliated Danyang Hospital of Nantong University, Jiangsu University, Zhenjiang, Jiangsu, China

**Keywords:** CGAS, colorectal cancer, inflammatory bowel disease, STING, therapy

## Abstract

The cyclic GMP-AMP synthase (cGAS)-stimulator of interferon genes (STING) signaling pathway is a critical mechanism of DNA sensing in innate immunity. Activation of this pathway can induce the production of interferons and proinflammatory cytokines. In the intestine, this pathway exhibits bidirectional regulatory properties, with appropriate activation maintaining homeostasis and inhibiting tumorigenesis, while excessive activation leads to inflammatory responses. A thorough exploration of the molecular mechanisms and regulatory networks of the cGAS-STING signaling pathway offers a significant theoretical foundation and potential treatment targets for developing novel strategies to treat intestinal diseases. This review summarizes the most recent developments on the function of the cGAS-STING regulatory pathway in colorectal tumors and inflammatory bowel disease. It discusses targeted therapeutic approaches that interfere with this pathway.

## 1 Introduction

The intestinal mucosal immune system is the core regulator of intestinal homeostasis. It achieves adaptive responses to commensal microorganisms and food-borne antigens by precisely balancing immune defense and immune tolerance. Disruption of this immune homeostasis can trigger chronic inflammatory responses and increase the risk of tumorigenesis (1–3). In recent years, the role of the innate immune system in the pathogenesis of intestinal diseases has attracted considerable attention. Among them, the cyclic GMP-AMP synthase (cGAS)-stimulator of interferon genes (STING) signaling pathway, as a key pathway for cytoplasmic DNA sensing, plays an important regulatory role in infection, inflammation, and tumorigenesis (4, 5). cGAS is a key molecule in cytoplasmic DNA sensing that can specifically recognize exogenous pathogens (such as viruses and bacteria) and endogenous damage-related (such as mitochondrial and nuclear sources) double-stranded DNA (dsDNA) (5, 6). Upon activation, it catalyzes the synthesis of 2'3'-cyclic GMP-AMP (cGAMP) from adenosine triphosphate (ATP) and guanosine 5'-triphosphate (GTP). It promotes the binding of cGAMP to STING, thereby cascadingly activating the nuclear factor-kappaB (NF-κB) and interferon regulatory factor 3 (IRF3) signaling pathways, eventually inducing the transcriptional expression of type I interferons and proinflammatory cytokines (7, 8).

With the recent expansion of studies on gut immune modulation, the cGAS-STING transduction pathway has become increasingly prominent in the pathophysiology of gastrointestinal conditions, including colorectal cancer (CRC) and inflammatory bowel disease (IBD). It is also worth noting that existing studies have shown that this pathway participates in the maintenance of intestinal homeostasis through a dual regulatory mechanism (9, 10). Therefore, thorough analysis of the molecular processes and networks that regulate the cGAS-STING axis in IBD and CRC will improve our understanding of the pathophysiology of these conditions and provide a basis for developing new, personalized treatment strategies (11, 12). However, the specific regulatory processes and translational applications of this route in intestinal diseases still face significant challenges. This review summarizes the latest advances in the role of the cGAS-STING signaling pathway in IBD and CRC and describes targeted therapeutic strategies targeting this signaling pathway.

## 2 The signaling pathway of cGAS-STING

### 2.1 DNA sensing by cGAS

DNA activates the cGAS-STING signaling pathway. When cGAS identifies exogenous or endogenous dsDNA, it promotes the synthesis of cGAMP. cGAMP activates a series of downstream signaling pathways, such as the type I interferon and NF-κB pathways, by binding to STING, thereby promoting inflammation (13, 14). cGAS-STING signaling has effects at the cellular level on autophagy, translation, metabolic homeostasis, cell concentration, DNA damage repair, aging, and cell death (15). Simultaneously, the cGAS-STING axis plays a crucial role in innate immunity and viral defense by detecting DNA (16).

### 2.2 STING activation and downstream signaling and effector functions

**Figure 1:**
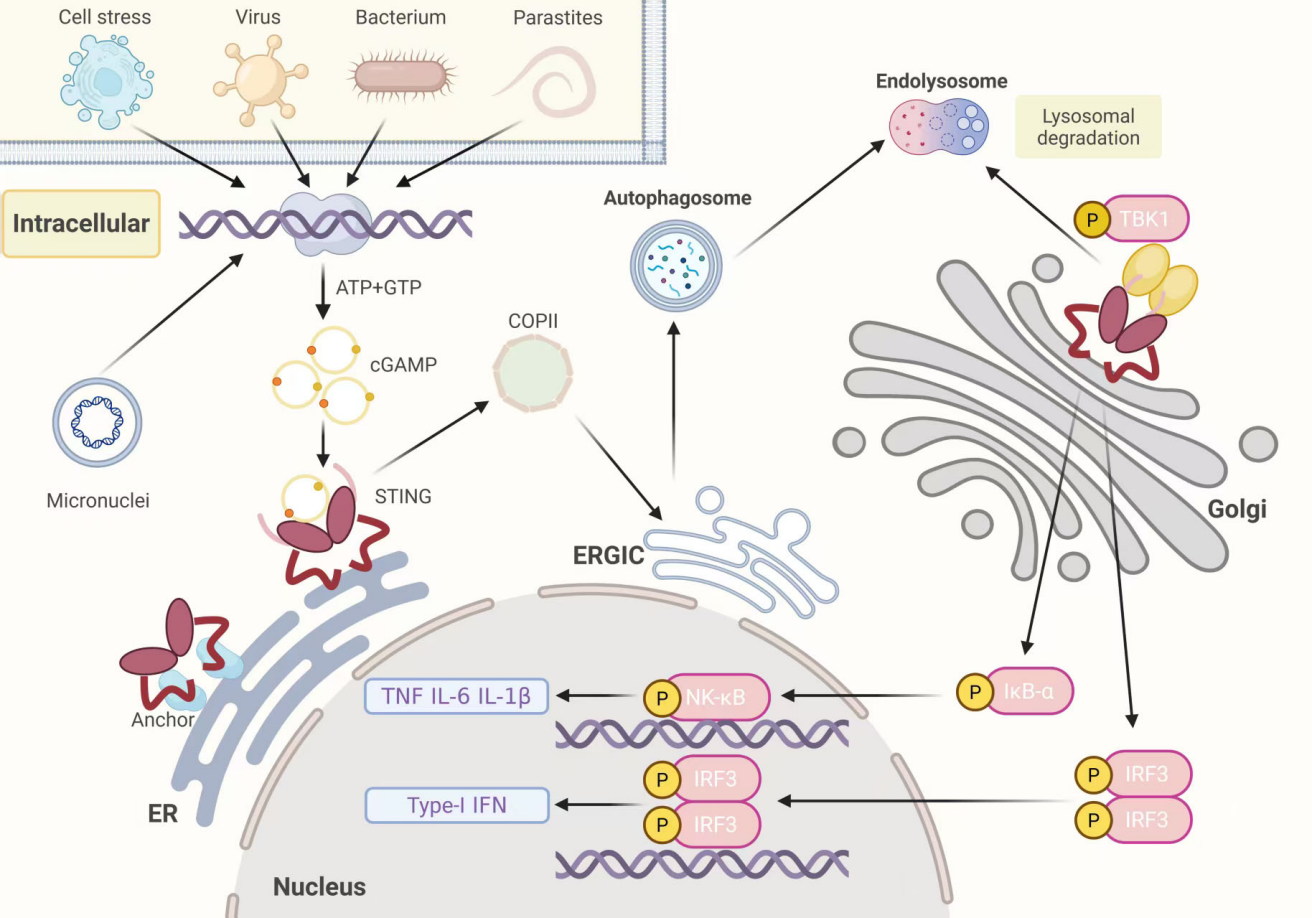
The cGAS-STING pathway's activation during innate immunological reactions. Following the identification of DNA from external and intracellular sources, cGAS dimers aggregate on dsDNA and catalyze the formation of 2',3'-cGAMP from ATP and GTP. cGAMP then binds to STING dimers in the ER, triggering STING oligomerization and releasing it from anchor factors. STING then enters COPII vesicles. As STING passes through the ERGIC and the Golgi apparatus, it attracts TBK1, promotes the transphosphorylation of STING by TBK1, and recruits IRF3. Consequently, IRF3 dimerizes and moves into the nucleus, inducing the release of type I interferons. STING activation also leads to the ubiquitination and breakdown of IκBα, releasing NF-κB. Following its translocation to the nucleus, NF-κB produces TNF, IL-6, IL-1β, and other chemicals. Finally, STING in the autophagosome and Golgi apparatus is transported to the lysosome for degradation. ATP, adenosine triphosphate; cGAMP, 2'3'-cyclic guanosine monophosphate-adenosine monophosphate; COPII, coat protein complex II; dsDNA, double-stranded DNA; cGAS, cyclic GMP-AMP synthase; ER, endoplasmic reticulum; ERGIC, ER-Golgi intermediate compartment; IL, interleukin; IκBα, inhibitor of nuclear factor kappa B alpha; IRF3, interferon regulatory factor 3; NF-κB, nuclear factor-kappaB; STING, stimulator of interferon genes; TBK1 serine/threonine protein kinase 1; TNF, tumor necrosis factor.

cGAS is a crucial enzyme implicated in sensing cytoplasmic DNA, possessing a nucleotide transferase domain along with two DNA-binding domains. As the critical functional domain of cGAS, the C-terminal nucleotidyltransferase (NTase) domain consists of a catalytic domain and two positive regions. When cGAS detects DNA from microbes such as viruses, retroviruses, or bacteria, or from self-DNA (17), the DNA ligand binds to cGAS in a minimum 2:2 combination, causing alteration in conformation in cGAS, which catalyzes ATP and GTP into 2',3'-cGAMP (16, 18, 19). At this point, a second messenger, cGAMP, attaches itself to the DNA-sensing hub STING, triggering a protein conformational change that leads to STING oligomerization into a tetramer. The endoplasmic reticulum (ER) transfers STING oligomers to the Golgi apparatus. Within the ER-Golgi intermediate compartment (ERGIC) of the Golgi apparatus, STING is palmitoylated and recruits serine/threonine protein kinase 1 (TBK1) (20). TBK1 transphosphorylates STING's C-terminal domain, recruiting the transcription factor IRF3 for stimulation, resulting in IRF3 dimerization and migration to the cell nucleus, inducing the release of type I IFN, with IFN-β being the primary type I IFN. Additionally, the STING signal can also phosphorylate IκBα (an NF-κB inhibitory protein) via TBK1 or the IKK complex, leading to its ubiquitination and breakdown, which releases NF-κB (21). Free NF-κBtranslocates to the cell nucleus, initiating the expression of cytokine genes, such as tumor necrosis factor (TNF), IL-6, and IL-1β (15, 17). After activation, STING is transported to the endolysosome for degradation (22). **Figure 1** illustrates the activity of the cGAS-STING pathway during innate immune responses.

### 2.3 Regulation of the cGAS-STING pathway

The cGAS-STING pathway is positively and negatively regulated by cellular components and enzymes, as well as additional DNA-sensing routes (9, 10). For example, the nucleic acid enzyme DNase II in lysosomes digests DNA in endosomes or autophagosomes, thereby preventing DNA from entering the cytoplasm and inhibiting cGAS activation. Three-prime repair exonuclease 1 (TREX1) is an exonuclease that degrades DNA in the cytoplasm. Defects in TREX1 are associated with various autoimmune and inflammatory diseases, including Aicardi-Goutieres syndrome and systemic lupus erythematosus, among others (23). Additionally, cGAS downstream ligands, like cGAMP and STING, can adversely affect cGAS activity by modifying it through processes such as sumoylation, phosphorylation, deubiquitination, glutamylation, and phosphodiesterase-catalyzed hydrolysis. Positive regulation of the cGAS-STING pathway is primarily achieved through post-translational cellular regulators via modification, direct interaction, or indirect assistance (4). **Table 1** summarizes the intracellular modulators of cGAS, cGAMP, and STING activity.

**Table 1:** Intracellular regulators that enhance or inhibit cGAS, cGAMP, and STING activity.

Target	Regulator molecule	Type of regulation	Modification method	Mechanism of action	Reference
cGAS	DNase II	Inhibition	Glycosylation	Degradation of DNA in endosomes/ autophagosomes to prevent leakage into the cytoplasm, which would lead to excessive activation of cGAS.	(23)
	TREX1 (DNaseIII)		Phosphorylation	Degradation of free dsDNA to prevent its activation of cGAS.	(24)
	SAMHD1		Phosphorylation/ tetramerization	Enhances MRE11 enzyme activity, degrades ssDNA at stalled replication forks, preventing autoinflammatory responses caused by cytoplasmic ssDNA accumulation.	(25)
	AIM2		Oligomerization	Forms inflammasomes, activates caspase-1 to degrade cGAS, and simultaneously induces pyroptosis to reduce intracellular DNA, inhibiting cGAS activation.	(26–28)
	Caspase-1		Proteolytic cleavage	1. Directly hydrolyzes cGAS protein after activation by inflammasomes. 2. Induces through activation of gasdermin D Indirectly inhibits cGAS and reduces DNA pyroptosis by inducing K^+^ efflux.	(28, 29)
	AKT		Phosphorylation	Phosphorylates the cGAS enzyme domain (Ser305/291 sites) to reduce its DNA-binding capacity.	(30)
	IFI16	Activation	Phosphorylation/acetylation	1. Detects viral DNA or damaged chromatin in the nucleus/cytoplasm, activating the STING-TBK1-IRF3 pathway. 2. Competitively binds to DNA, inhibiting excessive cGAMP production. 3. The HIN2 domain binds to the catalytic domain of cGAS, blocking its activity and maintaining immune homeostasis.	(31–34)
	TRIM41		Monoubiquitination	Binds to cGAS and promotes its monoubiquitination, aiding GAMP synthesis. Protects cGAS from autophagic degradation and enhances antiviral responses.	(35, 36)
	Mn²^+^ ions		Direct binding	Increases cGAS sensitivity to dsDNA and promotes STING activation.	(37)
cGAMP	ENPP1	Inhibition	Phosphodiesterase activity	Hydrolyzes the 2'3'-cGAMP termination signal to halt signaling.	(38)
	Zn²^+^ ions	Activation	Direct binding	Promotes cGAS-DNA phase separation, enhancing cGAS enzyme activity *in vitro* and in cells.	(39)
STING	AMFR	Inhibition	E3 ubiquitination	Promotes the transfer of STING from the ER to the Golgi apparatus, attracting IRF3 and TBK1	(40)
	MUL1		E3 ubiquitinylation	Drives STING vesicle transport from the ER to the Golgi apparatus.	(41)
	USP13	Activation	Deubiquitination	Targets the K63-ubiquitin chain of STING, blocking TBK1 recruitment and thereby inhibiting downstream signaling.	(42)
	ULK1		Phosphorylation (Ser366)	Promotes STING lysosomal degradation, consequentlypreventing sustained production of type I IFN.	(43)

STING, stimulator of interferon genes; DNA, deoxyribonucleic acid; cGAMP, 2'3'-cyclic GMP-AMP; dsDNA, double-stranded DNA; DNase II, deoxyribonuclease II; cGAS, cyclic GMP-AMP synthase; TREX1, three-prime repair exonuclease 1; SAMHD1, SAM domain and HD domain-containing protein 1; AIM2, absent in melanoma 2; AKT, protein kinase B; FI16, interferon-inducible protein 16; TRIM41, tripartite motif-containing protein 41; ENPP1, ectonucleotide pyrophosphatase/phosphodiesterase 1; AMFR, autocrine motility factor receptor; MUL1, mitochondrial E3 ubiquitin ligase 1; USP13, ubiquitin-specific peptidase 13; ULK1, Unc-51 like autophagy activating kinase 1.

### 2.4 The cGAS-STING pathway's various biological roles and disease implications

**Figure 2:**
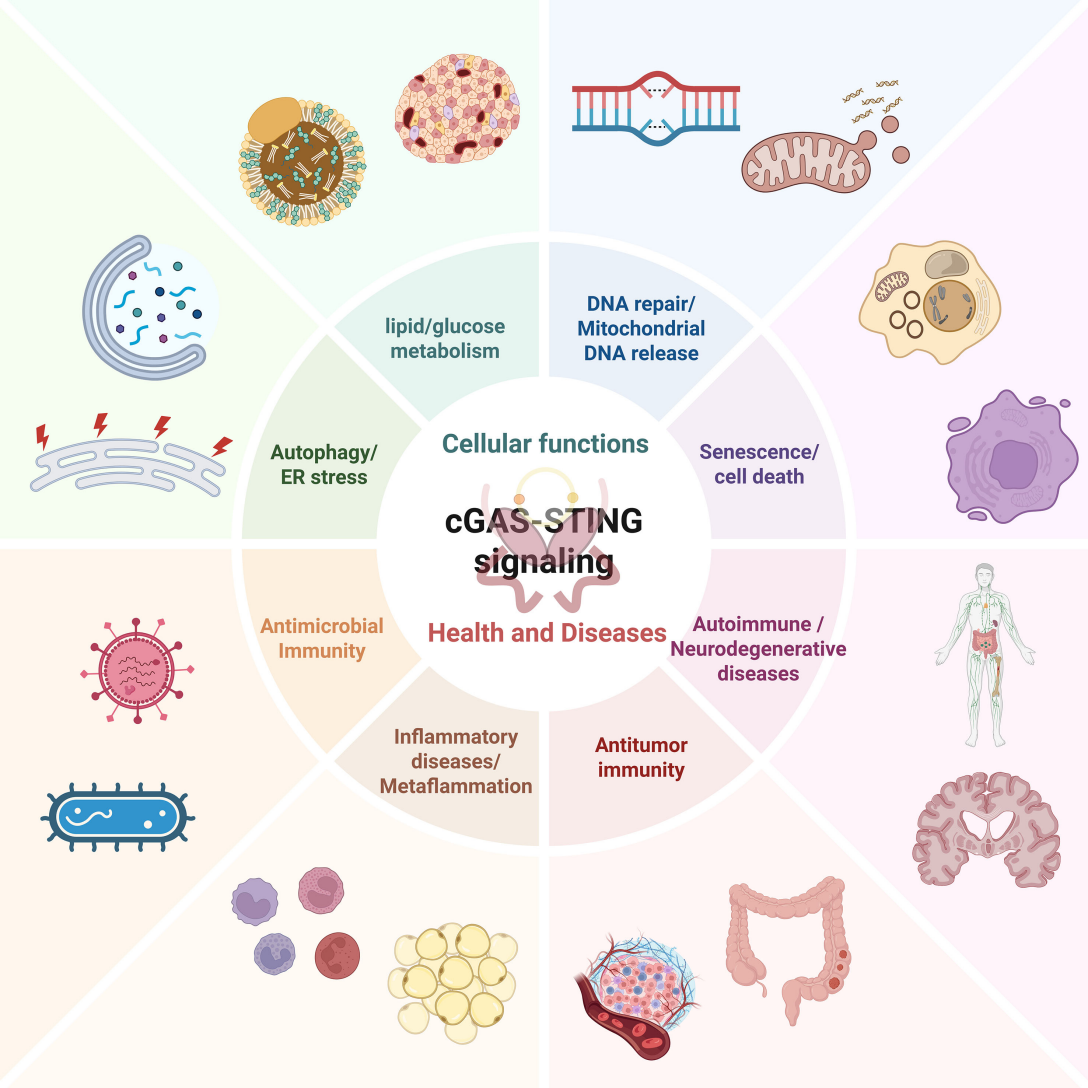
The cGAS-STING route in cellular homeostasis and diseases. The cGAS-STING route bidirectionally regulates cellular homeostasis and inflammation, balancing immune defense and disease onset. cGAS, cyclic GMP-AMP synthase; DNA, deoxyribonucleic acid; ER, endoplasmic reticulum; STING, stimulator of interferon genes.

The cGAS-STING route functions as the core molecular mechanism for cytoplasmic DNA sensing, contributing significantly to cellular homeostasis and immunological surveillance under physiological conditions. Dysfunction in this pathway is closely linked to the onset and progression of various conditions. Existing studies indicate that this signaling pathway not only mediates the classical IFN-I response but also participates in the control of multiple critical cellular biological processes through a multi-level molecular regulatory network. This pathway can trigger autophagy by activating TBK1 and autophagy-related proteins (such as ULK1 and Beclin-1) (44), thereby maintaining intracellular homeostasis and clearing pathogens or abnormal protein aggregates. Additionally, STING activation disrupts the ER membrane structure, inducing ER stress, and influences cell survival and death decisions through the unfolded protein response (UPR) (45). Furthermore, the signaling pathway of cGAS-STING can promote DNA damage repair by regulating repair factors, such as ataxia telangiectasia mutated (ATM) and ataxia telangiectasia and Rad3-related (ATR), contributing significantly to the preservation of genomic stability (46). Persistent activation of the cGAS-STING pathway may drive cellular senescence or programmed cell death through the p53/p21 or NF-κB pathways (46). Numerous studies have shown that the cGAS-STING signaling pathway plays a key regulatory role in the onset and progression of various diseases, including viral infections (47–49), metabolic endocrine disorders (50–52), autoimmune diseases (53–55), and neurological disorders (44, 55). **Figure 2** depicts how the cGAS-STING axis functions in cellular homeostasis and under various conditions.

## 3 The cGAS-STING signaling pathway in IBD

### 3.1 Overview of IBD

IBD is a chronic inflammatory disease of the gastrointestinal tract, primarily consisting of two subtypes: Crohn's disease (CD) and ulcerative colitis (UC) (56). Its clinical manifestations include gastrointestinal symptoms such as abdominal pain, diarrhea, fever, and rectal bleeding, as well as extraintestinal manifestations such as arthritis, osteoporosis, and psoriasis, and are often accompanied by cardiovascular diseases, metabolic syndrome, and cancer (57, 58). Epidemiological studies indicate that the incidence of IBD continues to rise, has gradually evolved into a global disease, and has severely impacted quality of life for patients (59). The development of IBD is affected by multiple factors, and recent research on its pathogenesis has made substantial progress, involving abnormalities in the gut microbiota, immune dysregulation, environmental changes, and genetic mutations. Numerous studies indicate that IBD's main cause is the interplay between microbes and the host. When the host has susceptible genes, it is prone to disrupting microbial homeostasis, allowing pathogens to invade the intestines, triggering destructive immune responses, inducing persistent inflammation, and promoting the progression of IBD (60). From an immunological perspective, Th17 cells are considered a primary pathogenic factor in IBD, playing a central role in the induction and maintenance of chronic intestinal inflammation in IBD patients (61). Th17 cells are extensively infiltrated in IBD patients' inflammatory intestinal mucosa, and the quantity of cells that release cytokines associated with Th17 and IL-17 is also increased in inflammatory tissues, in contrast to healthy tissues (8).

The gut mucosa gets exposed to both potentially harmful and commensal bacteria. The intestinal barrier serves as the first line of defense against intestinal microbiota. The gut microbiota is made up of immunological cells (such as intraepithelial lymphocytes, or IELs), differentiated epithelial cells and their secretory components (including mucins from goblet cells and antimicrobial peptides from Paneth cells), and stem cells (62). IECs are an indispensable component of the intestinal barrier, providing both physical and biochemical barriers to separate microorganisms from host tissues and maintain intestinal homeostasis. Homeostasis of the intestinal mucosa depends on the local immune system to maintain tolerance toward the normal microbiota and initiate effective immune responses to eradicate intestinal pathogens. An effective barrier function includes maintaining epithelial integrity, mucus layer production, and antimicrobial peptide secretion (63).

### 3.2 The dual role of cGAS-STING signaling in gut homeostasis and inflammation

**Figure 3:**
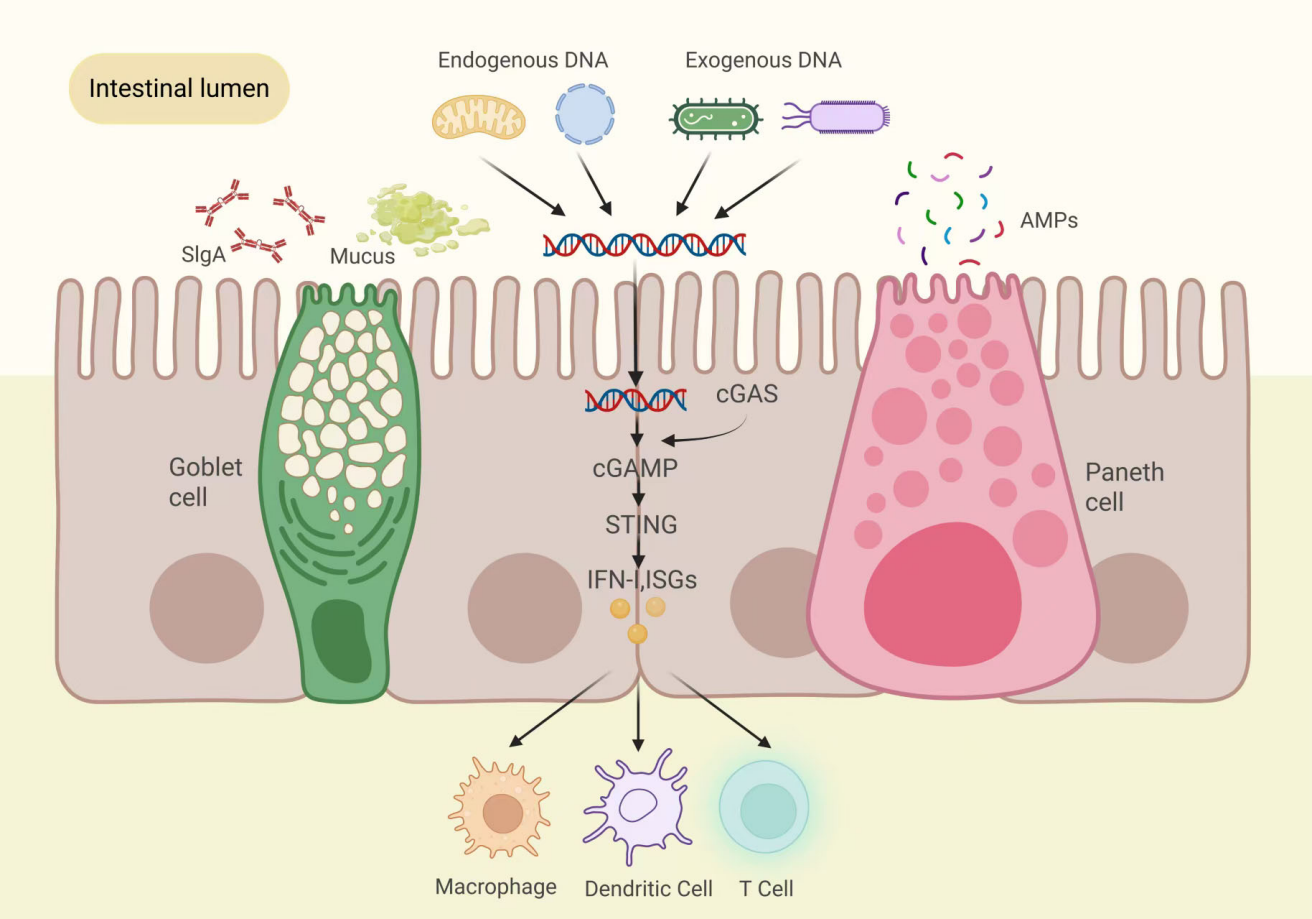
The function of cGAS-STING signaling activity in health. In a healthy environment, the symbiotic interaction between the gut’s microbes and the mucosa of the colon depends on the regulation of the endogenous STING pathway in host epithelial cells and innate immune cells. Activating the STING pathway significantly increases the expression of IFN-I and ISGs. This stimulation helps renew and maintain the integrity of the epithelial barrier, enhances the production of antimicrobial peptides by Paneth cells, boosts mucus production by goblet cells, and increases the synthesis of sIgA by plasma cells. Together, these effects synergistically strengthen the host's defense against pathogenic infections. This mutually beneficial relationship serves a crucial regulatory function in preserving immunological homeostasis and establishing intestinal microecological balance. DNA,deoxyribonucleic acid; cGAS, cyclic GMP-AMP synthase; cGAMP,2'3'-cyclic GMP-AMP; STING, stimulator of interferon genes; IFN-I, type I interferons; ISGs, interferon-stimulated genes; sIgA, secretory IgA; AMPs, antimicrobial peptides.

In a healthy environment, the interaction between gut microbes and the colon mucosa relies on the STING signaling pathway in both epithelial and immunological cells. Canesso and colleagues reported that, compared with control mice, systemic STING gene-knockout (STING^-^/^-^) mice showed reduced levels of TCRαβ-type IELs, goblet cells, mucins (MUC1 and MUC2), and secretory immunoglobulin A (sIgA) in the colon, which was accompanied by a marked alteration in gut microbial composition, characterized by an increase in pro-inflammatory bacteria (e.g., *Desulfovibrio*) and a decrease in beneficial taxa such as *Allobaculum* and *Bifidobacterium (*64). There was an increase in innate lymphoid cells 1 (ILC1) and ILC3 and a reduction in ILC2 and Treg cells in the lamina propria, indicating enhanced intestinal inflammatory responses and impaired immune regulatory function in these mice. Additionally, compared with the control group, these STING-deficient mice exhibited significantly increased susceptibility to colitis and *Salmonella typhimurium* infection. These findings collectively indicate that a STING deficit increases vulnerability to intestinal inflammation and bacterial infections in mice, underscoring STING's role as an important modulator of gut homeostasis (64). In intestinal immunity, stimulation of interferon (type I, IFN-I) secretion and interferon-stimulated genes (ISGs) enhances the epithelium barrier's regrowth and integrity (65, 66), encourages goblet cells to produce mucus, Paneth cells to produce antimicrobial peptides, and plasma cells to synthesize and secrete sIgA (9). This beneficial partnership is essential for maintaining the balance of immunological cells and fostering a healthy and harmonious intestinal environment (9). **Figure 3** illustrates the role of cGAS-STING signaling in maintaining immune homeostasis and establishing intestinal microbial balance.

**Figure 4:**
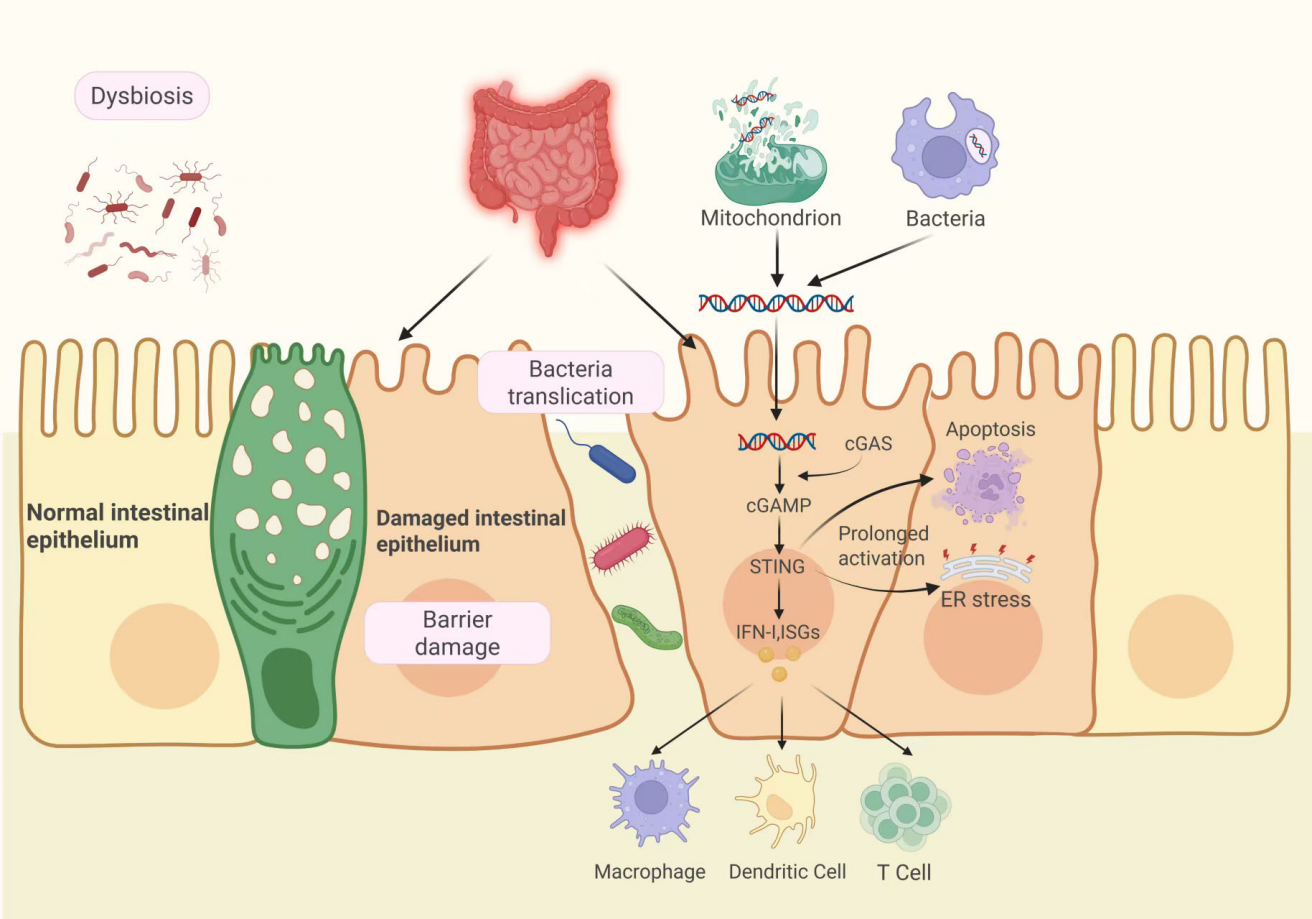
The function of cGAS-STING signaling activity in intestinal inflammation. In an inflammatory environment, dysbiosis of the microbiota and disruption of the epithelial barrier lead to the migration of harmful microbes into the LP. Both internal and external DNA stimulate the cGAS-STING transduction route in epithelial and immunological cells, promoting infiltration and activation of macrophages and inducing T cell aggregation and immune activation of dendritic cells (DCs), thereby exacerbating intestinal inflammatory responses. Additionally, STING activation and its downstream factors induce ER stress and apoptosis, further exacerbating barrier dysfunction. cGAS, cyclic GMP-AMP synthase; cGAMP,2'3'-cyclic GMP-AMP; ER,endoplasmic reticulum; IFN-I, Type I Interferons; ISGs, interferon-stimulated genes; LP, lamina propria; STING, stimulator of interferon genes.

Although STING plays a crucial function in preserving intestinal mucosal homeostasis, excessive activation of the cGAS-STING signaling pathway induces intestinal inflammation. IBD manifests as dysbiosis induced by epithelial barrier damage, with pathogenic microorganisms crossing the intestinal epithelial biological barrier and migrating into the lamina propria (LP), where they are recognized by immune cells, triggering local and systemic immune responses (8). Accumulating evidence suggests that cell-free DNA exerts a pro-inflammatory effect in IBD. Elevated mitochondrial DNA (mtDNA) levels in plasma have been consistently demonstrated in both dextran sulfate sodium (DSS)-induced murine colitis models and clinical cohorts of ulcerative colitis (UC) and Crohn's disease (CD) patients, with mtDNA concentrations showing significant positive correlation with disease severity (67, 68). Enhanced DNA damage and cytosolic DNA accumulation were consistently detected in colonic tissues from both experimental colitis models and IBD patients (69). Shmuel-Galia et al. (70), studying mice with STING gain-of-function Sting+/N153s (N153S), discovered that in contrast to the control category, the mice exhibited weight loss, shorter colon length, and compromised integrity of the intestinal barrier, specifically manifested by increased intestinal permeability, reduced ZO-1 levels, goblet cell loss and its byproducts (MUC2 and trefoil factor 3), decreased intestinal lymphocytes, and increased production of defensins and regenerating islet-derived protein 3-gamma. This indicates that the intestinal barrier in mice is severely damaged, immune responses are disrupted, and excessive activation of STING contributes to the onset and exacerbation of IBD. **Figure 4** shows the function of stimulation of cGAS-STING signaling in exacerbating intestinal inflammatory responses.

### 3.3 Extracellular vesicle-mediated DNA signaling and the cGAS-STING pathway in IBD

STING stimulation in immunological and epithelial cells can be triggered by cell DNA, cyclic dinucleotides (CDNs), and self-DNA from damaged cells, including nuclear (genomic) DNA (ncDNA) and mtDNA. These events can worsen intestinal mucosal inflammation (8, 9). Intracellular DNA (such as mtDNA or nuclear DNA) can bind to specific proteins, forming complexes that are encapsulated in microvesicles (mEVs) and then taken up by distant cells. Studies have shown that self-DNA from intestinal epithelial cells (IECs) can be transported via extracellular vesicles (EVs) to macrophages, activating the cGAS-STING signaling pathway and triggering inflammation in IBD (1, 71). Within the digestive system, EVs are primarily secreted by immunological cells and IECs. IEC EVs interact with dendritic cells (DCs), stimulating the maturation of DCs, Tregs, and macrophages with tolerogenic properties through immune regulatory signals, playing a crucial role in regulating intestinal mucosal and epithelial barrier function (72, 73). Microbial EV-host cell communication is abundant in intestinal mucosal tissues. Microbial-derived EVs can influence microbial composition and metabolism by transmitting signaling molecules, thereby regulating intestinal microbial balance and maintaining intestinal homeostasis. However, in the intestines of patients with IBD, EVs can facilitate communication between microbes and host cells, impacting the onset and progression of the disease (74, 75). Microbial DNA in extracellular vesicles (mEVs) is a primary cause of inflammation and damage to barrier functions. Gut microbiota-derived mEVs regulate inflammatory responses by entering host cells with bacterial products. Nie and the team studied IBD patients and colitis mice, discovering that microbial DNA in the gut is carried by mEVs that infiltrate the mucosa. This triggers the cGAS-STING signaling pathway, reducing intestinal barrier function and enhancing inflammatory responses (76). IBD patients exhibit significantly reduced complement receptor of the immunoglobulin family positive macrophages (CRIg+Mφ), allowing mEVs to diffuse into the mucosa (76). Blocking the cGAS-STING activation pathway reduces inflammation resulting from CRIg+Mφ deficiency and mEV leaking (74). Consequently, mEVs that harbor microbial DNA and the absence of CRIg+Mφ induce inflammation in IBD, with the cGAS-STING pathway being pivotal (76).

### 3.4 cGAS-STING pathway and autophagy interaction in IBD and intestinal epithelial homeostasis

Apart from promoting cytokine expression, numerous studies have demonstrated that STING activation can also trigger autophagy, a process that is crucial for the antimicrobial defense of the intestinal mucosa. The autophagy pathway can degrade misfolded proteins and protein aggregates as well as other damaged cellular components, thereby mediating the capture and killing of intracellular pathogens (77).cGAS can directly interact with the key autophagy protein Beclin-1, thus, activating the Beclin-1–phosphatidylinositol 3-kinase class III (PI3KC3) autophagy complex and inducing autophagy (78).Khan and colleagues found that cGAS promotes autophagy by upregulating Beclin-1, reduces IEC death, and plays an important role in maintaining intestinal epithelial homeostasis during human IBD and mouse colitis (79).Furthermore, upon binding to cGAMP, STING then translocates to the ERGIC and Golgi apparatus, where it recruits the PI3P effector protein WIPI2 and the ATG5-ATG12-ATG16L complex, providing a membrane source for LC3 lipidation (microtubule-associated protein 1A/1B-light chain 3B) and initiating non-canonical autophagy (77, 80). STING activation can also trigger endoplasmic reticulum stress, which in turn, negatively regulates the mTOR signaling pathway to induce autophagy (78). Interestingly, another study showed increased STING protein expression in a DSS-induced colitis mouse model (81). This observation further supports an association between STING upregulation and modulation of autophagy during intestinal inflammation. Further research indicates that autophagy can also disrupt STING signaling, thereby limiting STING-dependent IFN-I production. Typically, to prevent excessive activation of the cGAS-STING pathway, downstream signals are transiently activated, after which autophagolysosomes degrade cGAS-STING (82).In chronic intestinal inflammation and autophagy dysfunction caused by genetic factors, excessive STING signaling leads to more severe intestinal tissue damage and inflammation (83). STING signaling and autophagy interact and regulate each other to maintain cellular immunological homeostasis and antimicrobial defense.

### 3.5 cGAS-STING signaling and its role in cellular death pathways and IBD

Beyond its immunoregulatory functions, the cGAS-STING signaling pathway also participates in diverse cell death-related processes, encompassing cellular senescence, lysosome-dependent cell death (LCD), pyroptosis, apoptosis, and necroptosis. When DNA-containing bacteria invade cells, the cGAS-STING pathway becomes activated, leading to an IFN-I immune response. Excessive secretion of IFN-I overactivates the p53-p21signaling pathway elevatesp16INK4 levels, accelerating cellular senescence and inhibiting cellular function (84). The cGAS-STING signaling pathway upregulates NLRP3 expression through IRF3-mediated mechanisms. Activation of the NLRP3 inflammasome promotes the secretion of mature caspase-1 and IL-1β, ultimately triggering potassium efflux and pyroptosis (85). Notably, activated caspase-1 interacts with cGAS, thereby inhibiting IFN production (28). Butyrate alleviates the occurrence of Crohn's disease by inhibiting the cGAS-STING-NLRP3 axis-mediated pyroptosis in intestinal epithelial cells (86). Simultaneously, STING also contributes to lysosome-dependent cell death. Subsequent studies have shown that STING, after activating downstream signaling cascades, translocates into lysosomes, leading to lysosomal membrane permeabilization (LMP) and the leakage of lysosomal proteases into the cytoplasm (85, 87). However, the specific mechanisms remain unclear and require further investigation. In addition to pyroptosis, STING can directly bind phosphorylated TBK1, activating IRF3, which then binds to Bax and translocates to mitochondria, inducing cytochrome c release and thereby triggering apoptosis (88). Phosphorylated IRF3 can also activate caspase-8, cleave BCL-2, and induce Bax and Bak to promote apoptosis (88). Zhou et al. demonstrated that macrophage extracellular traps activate the cGAS-STING pathway, leading to enhanced apoptosis and reduced expression of tight junction proteins, thereby exacerbating DSS-induced colitis (89).Additionally, cGAS-STING senses intracellular DNA, signal through IFN-I and TNF receptors, activate RIPK3 in myeloid-derived macrophages, and subsequently induce necrotic apoptosis (90, 91). Thus, the cGAS-STING pathway activation promotes macrophage invasion and stimulation, as well as T cell accumulation in the LP, leading to a persistent imbalance of pro-inflammatory cells and cytokines in IBD, thereby exacerbating inflammatory damage.

### 3.6 ER stress and its impact on the cGAS-STING transduction pathway

Research indicates that numerous factors can influence the cGAS-STING signaling pathway (4). Among these, ER stress has garnered increasing attention in recent years. Disruption of ER homeostasis may impair STING signaling in IECs, weakening their anti-infective and inflammatory regulatory capabilities in the gut, and this leads to impaired immune defense function and the onset of IBD (92). ER stress refers to the excessive accumulation of misfolded or unfolded proteins within the ER (93). Intractable ER stress activates STING, and excessive STING signaling disrupts calcium balance, causing T cells to overreact to ER stress-induced responses, ultimately leading to cell death. This results in inflammation through the release of pro-inflammatory cytokines, impaired antimicrobial defense, or induced cell death (94–96). Becker and colleagues found that ER stress promotes cellular regulation of amino acid transport and mitochondrial 1C metabolism to enhance redox balance, thereby maintaining cellular proliferation and immune function (97). Chronic ER stress-induced glutathione (GSH) metabolic remodeling serves a key role in the antioxidant and viral immune responses of IEC (90). However, prolonged stress leads to depletion of the antioxidant system, impairing the cGAS-STING activation immune pathway and increasing susceptibility to viral infections (e.g., Cytomegalovirus (CMV)) (97). Inhibiting ROS accumulation induced by ER stress restores STING activity and antiviral responses. This study demonstrates the potential clinical value of antioxidant therapy in intestinal inflammation and viral control (97).

In summary, STING is essential for preserving the homeostasis of the intestinal mucosa. However, excessive activation of the cGAS-STING signaling pathway can lead to inflammatory responses, exacerbate intestinal tissue damage, and encourage the onset of IBD. Notably, in addition to the cytoplasmic discharge of self-DNA due to cellular damage or stress, microbial DNA transmitted by extracellular vesicles (mEVs) is also an important cGAS-STING pathway inducer. STING signaling-induced autophagy plays an important role in the antimicrobial defense of the intestinal mucosa, and conversely, autophagy can also interrupt STING signaling to prevent its excessive activation. Additionally, the cGAS-STING pathway is involved in cell death pathways, including LDCP, apoptosis, and necroptosis. It is also known that the cGAS-STING signaling pathway is influenced by various factors (such as DNA properties, regulatory proteins, viral interference, and cellular state). Further research into these factors and their mechanisms may uncover new clinical applications.

## 4 The cGAS-STING signaling pathway in CRC

CRC is among the most prevalent cancerous growths worldwide, ranking second in cancer-related mortality and third in prevalence among all cancerous tumors, presenting a major risk to human life and well-being (98). CRC arises from tumor stem cells or cells that have stem cell-like properties. The development of CRC is driven not only by the accumulation of genetic alterations in cancer cells but also by immunosuppressive conditions within the tumor microenvironment (99). Studies have shown that reactive nitrogen and reactive oxygen species generated by inflammatory cells can cause alterations in important genes implicated in tumorigenesis. Additionally, specific inflammatory triggers, such as NF-κB and cyclooxygenase, are essential to the carcinogenic process (100). The cGAS-STING pathway influences CRC by regulating the intestinal epithelial barrier and enterocyte proliferation. Experimental studies in mice demonstrate that cGAS deficiency compromises epithelial barrier integrity, worsens inflammation, and increases tumor burden through STAT3 activation and immunosuppression (101). Moreover, STING deficiency elevates pro-inflammatory cytokine production, decreases IL-18 and IL-22 regulation, and impairs tissue repair, thereby promoting CAC progression (102). Consequently, chronic IBD can increase the risk of developing CRC (100).

**Figure 5:**
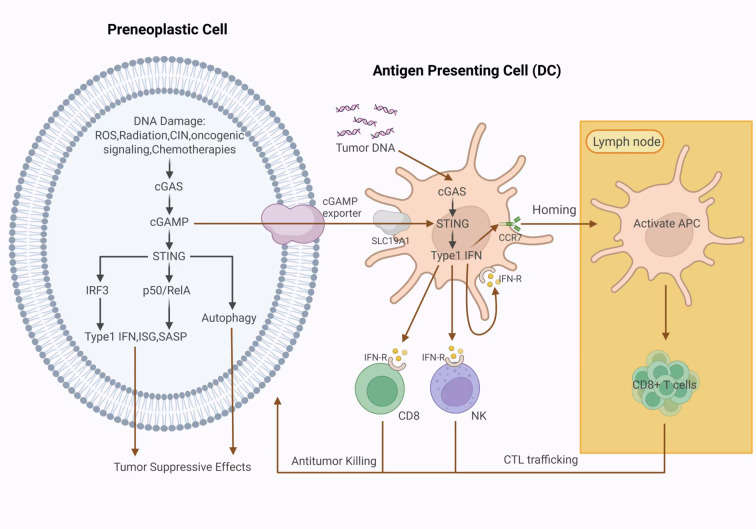
Role of STING pathway in tumor suppression. In early tumor precursor cells, the cGAS-STING pathway functions as a tumor suppressor, counteracting the carcinogenic effects induced by DNA damage. In tumor cells, cGAS can recognize cytoplasmic DNA that is produced by various sources of DNA damage. Activation of the cGAS-STING pathway upregulates the expression of type I interferons, ISGs, and SASP genes, as well as autophagy, thereby mediating tumor suppression. Furthermore, the cGAS-STING signaling pathway regulates anti-tumor immunological reactions by facilitating the interactions between immunological and tumor cells in the TME. cGAMP released by tumor cells and tumor-derived DNA can be taken up by APCs (such as DCs), thereby activating thecGAS-STING pathway, triggering the secretion of pro-inflammatory cytokines and IFN-I, and ultimately activating the tumor-killing functions of effector immunological cells, including NK and CD8+ T cells. This IFN-I-dependent mechanism significantly enhances the expression of CCR7 in APCs and their capacity for lymphatic migration, thus improving their efficiency in homing to draining lymph nodes. APC, antigen-presenting cell; DC, dendritic cells; DNA, deoxyribonucleic acid; ROS, reactive oxygen species; CIN,chromosomalinstability; cGAS, cyclic GMP-AMP synthase; cGAMP, 2'3'-cyclic GMP-AMP; STING, stimulator of interferon genes; IRF-3, interferon regulatory factor 3; IFN-I, Type I Interferons; ISGs, interferon-stimulated genes; SASP, senescence-associated secretory phenotype; SLC19A1, Solute carrier family 19 member 1; CCR7, C-C chemokine receptor type 7; TME, tumor microenvironment; NK, natural killer.

Several investigations have shown that the signaling of cGAS-STING is essential for preventing the growth of tumors and maintaining the effectiveness of anti-tumor therapy. Activating the cGAS-STING signaling pathway could serve as a potential new therapeutic strategy for CRC. ThecGAS-STING pathway regulates various aspects of the cancer immune cycle, including tumor antigen release, antigen presentation, T cell activation, T cell transport and infiltration into tumor tissue, and T cell recognition and killing of tumor cells, exerting either anti-tumor or pro-tumor effects (103).Its activation can enhance tumor antigen presentation, promote the infiltration of effector T lymphocytes, and synergize with PD-1/PD-L1 immune checkpoint inhibitors (104).The stimulation of the cGAS-STING axis in cancerous cells usually arises from genetic instability or failures in DNA repair. Nuclear DNA leaking and development of extra-nuclear micronuclei are important mechanisms for cGAS activation. When micronuclei rupture, the DNA enclosed within them enters the cytoplasm (14), where cGAS binds to this DNA, catalyzing the synthesis of cGAMP and activating the STING pathway (105). As early as 2014, prior work by Woo et al.experimentally demonstrated that tumor-derived cytoplasmic DNA activates the host's cGAS-STING pathway, promoting IRF3-dependent IFN-β production, thereby stimulating the maturation of innate immune cells and promoting the development of an inflammatory milieu around a tumor (106). Both studies also indicated that mice lacking STING showed increased susceptibility to various malignant tumors and decreased survival rates, further highlighting the critical function of STING-mediated immunological reactions in the inhibition of tumors. Additionally, Ohkuri and team found that local application of STING agonists (c-di-GMP) amplifies IFN-I signaling and enhances cellular immunological reactions, demonstrating the role of STING agonists in anti-glioma immunotherapy and providing guidance for their clinical application in combination with tumor antigen vaccines (107). Notably, it was also found that DCs can phagocytose cGAMP derived from surrounding cells or tumor cells. The ingested cGAMP can be transported from endosomes to the cytoplasm, triggering the STING signaling cascade within DCs (108). In summary, STING stimulation promotes DCs, macrophages, CD8+ T cells, and NK cells to infiltrate, activate, proliferate, and cross-prime. This dual stimulation cascade effectively inhibits carcinogenesis by promoting pro-inflammatory immune responses in the tumor microenvironment (9). **Figure 5** illustrates the function of the STING pathway in tumor suppression.

Numerous studies have demonstrated that the cGAS-STING pathway plays a pivotal role in CRC. cGAS-expressing cancer cells can identify cytoplasmic DNA and produce cGAMP (109), which can induce the STING pathway to stimulate the secretion of TNF-α and IFN-β, leading to substantial tumor cell necrosis (110).The mechanism of DNA mismatch repair (MMR) helps maintain DNA stability (100). During DNA damage, the MMR system facilitates cell cycle arrest and apoptosis, and its inactivation contributes to cancer (100).Previous research by Kaneta et al.analyzed public data and clinical tissue samples and found that the cGAS-STING signaling pathway is highly expressed in tumor cells of defective mismatch repair (dMMR)/microsatellite instability (MSI) CRC, and the stimulation of this process promotes the recruitment of CD8+ tumor-infiltrating lymphocytes and enhances the immune microenvironment (111). Experiments also showed that increased cGAS and STING expression in dMMR/MSI CRC is related to tumor immune activity and a better prognosis, and that downregulating the DNA repair gene MLH1 increases cGAS-STING pathway activation. This suggests that STING agonists may be suitable for treating CRC, and the high expression of the cGAS-STING pathway in dMMR/MSI CRC may provide a potential target for future immunotherapy (111). Nakajima and team found that approximately 60% of proficient DNA mismatch repair (pMMR) CRC cases simultaneously lack cGAS and STING expression (cGAS-/STING-), particularly in advanced tumors where STING expression is markedly reduced, possibly due to histone methylation regulation of the STING promoter region (112). Less than 10% of patients with pMMR and microsatellite stability (MSS) show cGAS+/STING+ expression, which correlates with increased infiltration of CD8+ and CD4+ T cells. Thus, cGAS-STING expression in tumor cells could be a useful indicator to forecast the success of immune checkpoint inhibitor (ICI) therapy in patients with pMMR/MSS CRC (112). Additionally, autophagy triggered by cGAS-STING stimulation can prevent the alteration of healthy cells into cancer cells by inducing cell death mediated by autophagy. This process responds to abnormal mitosis in healthy cells, eliminating cells that may undergo transformation and thereby protecting the body from the threat of carcinogenesis (113). However, the cGAS-STING axis enables tumor cells to avoid immunological detection. Certain cancer cell lines have STING and cGAS promoters that are susceptible to loss-of-function mutations or epigenetic silencing, which inhibits the cGAS-STING pathway (113).

As mentioned earlier, EVs can mediate communication between intestinal cells, maintain the gut mucosa barrier, participate in inflammatory processes, and regulate immune responses. Recently, mounting data have demonstrated that tumor-derived EVs also contribute significantly to the cGAS-STING regulatory cascade (114). Due to their small size, inherent biocompatibility, high physical and chemical stability (115), long-distance communication capabilities (115), and ease of interaction with cells (116), we can utilize gene engineering, metabolic labeling, and exogenous delivery technologies to modify EVs, which could open new avenues for nanomedicine (117). Exosomes are a specific subtype of EVs. Diamond et al. found that tumor cells secrete exosomes rich in ENPP1, which can hydrolyze extracellular cGAMP and cGAMP bound to LL-37, thereby inhibiting the cGAS-STING signaling pathway in immune cells (118). Using specific ENPP1 inhibitors can restore cGAS-STING activity and enhance anti-tumor immunity (114, 118). EVs are naturally occurring small vesicles that can deliver STING agonists such as cyclic dinucleotides to specific cells, including immune cells within the TME. STING agonists utilize the body's immunological system to identify and destroy cancer cells, playing a crucial role in cancer therapy. This approach not only enhances the efficacy of STING stimulation but also reduces systemic toxicity (119). ExoSTING is an exogenous cyclic dinucleotide-loaded engineered EV that can utilize the inherent ability of EVs to facilitate communication between APCs and tumor cells in the TME, directly delivering STING agonists to the TME to enhance local immune effects. Its key feature is prolonged retention in tumors, where it initially stimulates TME APCs, promoting local Th1 reactions, CD8+ T recruitment, and systemic defense against tumors, while reducing systemic inflammation. ExoSTING's precise delivery enhances drug targeting, improves stability and circulation time, reduces immune rejection and tolerance, and offers a therapy with a broader safety margin for cancer treatment (120).

Overall, the cGAS-STING axis inhibits the growth of tumors in CRC, and drugs that activate or modulate the cGAS-STING axis are being explored as a potential therapeutic strategy for CRC. Additionally, it is found that using EVs for the precise delivery of STING agonists can enhance local immune effects while reducing side effects, demonstrating the application potential of nanomedicine in drug delivery and immune regulation.

## 5 Treatment of IBD and CRC through targeting the cGAS-STING pathway

### 5.1 IBD

IBD, as a global disease with rapidly rising incidence rates, not only severely threatens human health but also imposes a heavy burden on individuals, families, and society (121). Conventional drug therapies are typically used to manage IBD, such as aminosalicylates (122), corticosteroids (123–126), immunomodulators (127, 128), and biologics (129). In certain instances, surgery is employed when necessary (130). In recent years, new therapies for IBD have surfaced, including monoclonal antibody therapy (131), microbiome modulation strategies (132), stem cell transplantation (133), and exosome treatment (134). However, these therapies are still in the research phase and face many challenges. Despite the availability of various treatment options for IBD, treatment outcomes remain suboptimal, necessitating the urgent development of new therapeutic approaches (92).

Targeted therapy has been widely adopted and represents a highly promising treatment strategy (135, 136). Considering the significance of the cGAS-STING signaling route in intestinal homeostasis and IBD, addressing it may provide a novel treatment approach. Ma et al. observed that Gasdermin D (GSDMD) is a negative regulator of the cGAS-STING signaling pathway in macrophages, and pharmacological inhibition of cGAS can reverse IBD symptoms in GSDMD-deficient mice (137). This confirms that GSDMD can control inflammation by inhibiting the cGAS-STING pathway, indicating that focusing on the GSDMD-cGAS signaling cascade may have potential value in the management of IBD (137). Additionally, developing novel oral nanomedicines by targeting the cGAS-STING signaling pathway has become a new direction in IBD treatment. Guilbaud and colleagues encapsulated the cGAS-STING inhibitor H-151 in lipid nanocapsules. This encapsulation induced the secretion of glucagon-like peptide-2 (GLP-2) and selectively targeted the cGAS-STING pathway along with its key downstream markers. As a result, it inhibited the expression of pro-inflammatory cytokines, promoted mucosal repair, and significantly reduced inflammatory responses in a mouse model of colitis. This method is highly selective, cost-effective, and scalable, offering great clinical application potential (138).

### 5.2 CRC

Numerous studies have highlighted the growing importance of the cGAS-STING signaling pathway in CRC, with targeting this pathway in CRC increasingly becoming a hot topic. The proper stimulation of immune cells' cGAS-STING pathway can inhibit tumor growth, while sustained activation may aid carcinogens in inducing tumor formation and blocking T cell-driven adaptive immunity (139). Chemotherapy and radiotherapy are well known for their effectiveness in halting the progression of cancer. Drugs that target and modulate this pathway may work in tandem with cGAS-STING agonists to reverse chemotherapy/radiotherapy resistance and improve clinical efficacy (3). Zhu et al. found that knocking down death-associated protein (Daxx) enhances the anti-tumor effects of chemotherapy drugs, while overexpressing Daxx reduces chemotherapy sensitivity (140). Additionally, Daxx inhibits the cGAS-STING activation pathway, weakening chemotherapy-induced immunogenic cell death (ICD) and anti-tumor immune reactions, thereby impairing the efficacy of chemotherapy in CRC. Targeting the Daxx/cGAS-STING axis could be a therapeutic strategy to improve chemotherapy efficacy in CRC patients (140). Li and colleagues demonstrated that phosphatidylinositol-3,4,5-trisphosphate RAC exchanger 2 (PREX2) overexpression is associated with CRC radiation resistance. PREX2 enhances DNA repair capacity by upregulating DNA-protein kinase, catalytic subunit (PKcs), prevents the induction of the radiotherapy-induced STING-IRF3-IFN signaling axis, and simultaneously weakens radiotherapy-induced ICD and CD8+ T cell infiltration (141). Additionally, targeting PREX2 inhibitors was found to reverse radiation resistance, enhance radiation sensitivity, and restore activation of the cGAS-STING-IFN pathway (141). In summary, PREX2 can be used as a marker to determine therapeutic targets and radiation therapy efficacy in CRC, and combining it with STING agonists can further synergistically enhance radiation therapy effects, providing a new direction for overcoming radiation resistance in CRC (141).

In addition to chemotherapy and radiotherapy, immunotherapy is emerging as a groundbreaking therapy modality, including ICIs, cancer vaccines, chimeric antigen receptor T-cell therapy, and tumor-infiltrating lymphocytes. For CRC, ICIs serve as the primary form of immunotherapy for populations with high MSI and dMMR (99). However, their effectiveness is limited in MSS populations (99). Notably, Duan and colleagues revealed that drug inhibition or gene knockout of protein arginine methyltransferase 6 (PRMT6) induces an MSI phenotype in MSS-CRC, characterized by MMR deficiency (142). PRMT6 deficiency causes increased infiltration of immunological cells, including NK and CD8+ T cells. Long-term inhibition of PRMT6 leads to the accumulation of cytoplasmic DNA, which activates the cGAS-STING route, thereby boosting anti-tumor immune responses (142). Additionally, it was found that combining PRMT6 inhibitors with programmed cell death protein 1 (PD-1) antibodies enhances sensitivity to immune checkpoint blockade therapy, significantly improving treatment efficacy and prolonging survival time in animal models (142). Targeting PRMT6 converts MSS-CRC into an MSI-like state, enhancing the effectiveness of immunotherapy and offering new insights and strategies for CRC treatment (142). Luo et al. discovered that gasdermin E (GSDME)-induced damage to the mitochondria activates the cGAS-STING-IFNβ route, leading to increased CD8+ T cell infiltration, which can synergize with ICIs, providing a potential new target for CRC treatment (143). Wu et al.demonstrated *in vitro* that RC48 not only induces cell cycle arrest but also significantly inhibits the proliferation of HER2-positive CRC by alleviating HER2-mediated immune suppression and turning on the cGAS-STING regulatory cascade, thus augmenting tumor susceptibility to anti-PD-1 immunotherapy (144). Furthermore, *in vivo* mouse models confirmed that the combination of RC48 with anti-PD-1 therapy significantly inhibits tumor growth and enhances anti-tumor immune responses, offering a new treatment option for HER2-positive CRC (144). As noted by Liu's research group, the synergistic effect of radiotherapy and ATR inhibitors (such as berzosertib) can stimulate the cGAS-STING-pTBK1/pIRF3 pathway by inhibiting SH2 domain-containing phosphatase 1 (SHP1) function, promoting T cell infiltration in colorectal tumors, transforming cold tumors into hot tumors, and improving immunity against tumors (145). Additionally, the combination of radiotherapy, berzosertib, and PD-1 inhibitors was found to significantly improve treatment outcomes in MSS-CRC, potentially offering a new therapeutic strategy for MSS-CRC (145). Lovastatin is a widely utilized hypolipidemic agent. Huang and colleagues found that lovastatin induces mitochondrial oxidative stress in human CRC cells, resulting in the cytoplasmic release of mtDNA, which triggers the cGAS-STING signaling pathway in HCT116 cells, ultimately inducing apoptosis and inhibiting the growth and proliferation of CRC cells (146). This study provides new potential targets and a theoretical basis for CRC treatment, as well as experimental support for the application of lovastatin in the field of anticancer therapy (146). The treatment approaches that alter the cGAS-STING pathway in IBD and CRC are summarized in **Table 2**.

**Table 2:** Targeted therapeutic strategies modulating cGAS-STING pathway in IBD and CRC.

Target	Condition	Intervention	Mechanism	Effect	Reference
GSDMD- cGAS axis	DSS-induced colitis (IBD)	RU.521 (cGAS inhibitor)	1.GSDMD deficiency leads to hyperactivation of cGAS-STING pathway 2.PharmacologicalcGAS inhibition reduces STING-dependent inflammation	Improves clinical symptoms and histopathological damage of colitis	(137)
cGAS-STING signaling	Experimental colitis	H-151-loaded lipid nanocapsules	1.Oral nanocarriers enhance colonic H-151 delivery 2.Induces GLP-2 secretion and strengthens epithelial barrier	Reduces pro-inflammatory factors and promotes intestinal mucosal barrier repair	(138)
Daxx/STING interaction	Chemoresistant CRC	Daxx siRNA + Oxaliplatin	1.Daxx directly binds cGAS 2. Knockdown enhances STING-dependent ICD	Enhances chemotherapy sensitivity and promotes anti-tumor immune response	(140)
PREX2-DNA-PKcs pathway	Radioresistant CRC	NCT-503 (PREX2i) + X-ray	1. PREX2 high tumors show impaired STING activation 2.Inhibition restores DNA damage-induced STING signaling	Reverses radioresistance and enhances CD8+ T cell tumor infiltration	(141)
PRMT6-mediated MMR dysregulation	MSS-CRC	EPZ020411 + anti-PD-1	1.PRMT6 KO induces MSI-like phenotype 2. Activates STING-TBK1 axis	Improves immune checkpoint blockade efficacy and prolongs survival	(142)
GSDME-dependent mtDNA release	Immunogenic CRC	GSDME overexpression + anti-PD-L1	1.Mitochondrial damage → mtDNA release 2.ActivatescGAS-STING-IFNβ axis	Enhances immunotherapy response and produces synergistic anti-tumor effects	(144)
HER2-STING crosstalk	HER2+ CRC	RC48 + nivolumab	1.RC48 downregulates pHER2 2.Activate STING pathway	Improves PD-1 antibody therapeutic effect and inhibits tumor progression	(144)
ATR/SHP1/STING axis	MSS-CRC	Berzosertib + RT + pembrolizumab	1.Blocks SHP1-mediated STING dephosphorylation 2.Enhances antigen presentation	Converts immunologically "cold" tumors and improves PD-1 treatment response rate	(145)
mtDNA-STING pathway	CRC cells	Lovastatin	1. Induces mtROS and mtDNA release 2.Activates caspase-3-dependent apoptosis	Induces tumor cell apoptosis and inhibits tumor proliferation	(146)

cGAS, cyclic GMP-AMP synthase; STING, stimulator of interferon genes; cGAMP, 2'3'-cyclic GMP-AMP; DNA, deoxyribonucleic acid; CDN, cyclic dinucleotide; IBD, inflammatory bowel disease; CRC, colorectal cancer; GSDMD, gasdermin D; DSS, dextran sulfate sodium; GLP-2, glucagon-like peptide-2; Daxx, death domain-associated protein 6; PREX2, phosphatidylinositol-3,4,5-trisphosphate RAC exchanger 2; PRMT6, protein arginine methyltransferase 6; MMR, mismatch repair; MSI, microsatellite instability; MSS, microsatellite stable; GSDME, gasdermin E; mtDNA, mitochondrial DNA; ATR, ataxia telangiectasia and rad3-related protein; SHP1, SH2 domain-containing phosphatase 1; mtROS, mitochondrial reactive oxygen speciess; ICD, immunogenic cell death; siRNA, small interfering RNA; RT, radiation therapy; pHER2, phosphorylated HER2; KO, knockout; PKcs, protein kinase, catalytic subunit; PD-1, programmed cell death protein 1.

## 6 Classification and mechanisms of cGAS-STING inhibitors and agonists

### 6.1 cGAS-STING inhibitors

Given the dual role of the cGAS-STING pathway in the pathogenesis and immune surveillance of IBD and CRC, appropriate targeted stimulation during disease progression can restore intestinal homeostasis and enhance anticancer immunity, demonstrating significant potential for clinical application. Inhibitors that downregulate the cGAS-STING pathway can mitigate the progression of autoimmune disorders and localized inflammation, and agonists that upregulate the cGAS-STING pathway can enhance immune system reactions and limit the entry of extracellular infections (4). Multiple studies have shown that inhibitors targeting the cGAS-STING signaling pathway play a key role in regulating innate immune responses. Recently, researchers have developed various specific inhibitors due to a better understanding of the molecular mechanisms involved in this pathway. Based on their target sites, these inhibitors can be categorized into two main classes: cGAS and STING inhibitors. cGAS inhibitors consist of DNA-competitive inhibitors and catalytic site inhibitors, while STING inhibitors include CDN-binding/conformation-regulating and STING palmitoylation modification inhibitors (147). As shown in **Table 3**, these drugs inhibit the cGAS-STING signaling axis through various mechanisms, offering new intervention targets for the future treatment of related diseases.

**Table 3:** cGAS or STING inhibitors.

Target	Type	Name	Mechanism	Effect	Reference
cGAS	Catalytic site inhibitor	PF-06928215	Occupies the cGAS catalytic site, competitively blocks ATP/GTP substrate binding, and inhibits cGAMP synthesis	Reduces IFN-1 and inhibits the release of pro-inflammatory factors	(148)
		Aspirin	Acetylates the cGAS residues Lys384, Lys394, and Lys414, blocking its ability to bind to DNA	Inhibits cGAS activation, treating autoimmune diseases	(149)
		G150	Directly binds to the cGAS catalytic pocket, competitively blocking ATP/GTP substrate binding	Inhibits cGAMP synthesis in human primary macrophages, suppressing the innate immune response	(150)
	DNA competitive inhibitor	Hydroxychloroquine	Embeds into the phosphate backbone of dsDNA, competitively inhibiting cGAS binding to DNA	Reduces immunosuppressive cytokines	(151)
		Suramin	Competitively binds to DNA, blocking its interaction with cGAS and inhibiting cGAMP synthesis	Inhibits tumor-associated inflammation and enhances anti-tumor immunity	(152)
STING	CDN binding/conformation regulation	H-151	Competitively connects to STING's CDN binding site	Inhibits STING-dependent TBK1-IRF3 phosphorylation and IFN-β production	(153)
		AstinC	Selectively interacts with the STING's C-terminal region, blocking IRF3 recruitment into the STING-TBK1 signaling complex	Treats autoimmune inflammatory diseases	(154)
		Nitrofurans	Binds to Cys91 to suppress STING palmitoylation	Enhances STING-dependent IFN-β production, promoting immunological stimulation in the TME	(155)
		NO2-FAs	Covalently alter the Cys91 and Cys88 residues of STING to suppress its activation	Regulate innate immunity to treat inflammatory diseases that depend on the STING pathway	(156)

cGAS, cyclic GMP-AMP synthase; STING, stimulator of interferon genes; cGAMP, 2'3'-cyclic GMP-AMP; DNA,deoxyribonucleic acid; CDN, cyclic dinucleotide; NO2-FAs, nitro-fatty acids; ATP, adenosine triphosphate; GTP, guanosine triphosphate; Cys, cysteine; TBK1, TANK-binding kinase 1; IRF3, interferon regulatory factor 3; IFN-β, interferon-beta; TME, tumor microenvironment.

### 6.2 cGAS-STING agonists

The cGAS-STING pathway is a central element of the innate immunological system, and its activation can induce strong IFN-I and pro-inflammatory cytokine responses, thereby enhancing anti-tumor immunity. In recent years, agonists of this pathway have emerged as a research hotspot due to their potential in tumor immunotherapy (147). These agonists can be classified into cGAS agonists and STING agonists based on their target sites. **Table 4** summarizes the mechanisms of representative molecules and their functional roles in anti-tumor immunity. Since activating STING can initiate 2'3'-cGAMP, most STING activators are mimics of 2'3'-cGAMP synthesis, many of which undergo chemical changes to enhance their stability and resistance to hydrolysis. These modifications can extend their half-life *in vivo*, more effectively stimulate the STING pathway, activate immune responses, and thereby enhance anti-tumor effects (15). Such modified synthetic cGAMP holds significant potential in immunotherapy research and development (167). Previous studies have shown that the STING activator cGAMP plays a key role in activating the cGAS-cGAMP-STING-IRF3 pathway for anti-tumor effects. Li et al. found that cGAMP can promote cytokine production, activate dendritic cells, and drive T cell-mediated immune responses, demonstrating significant anti-tumor effects in a mouse colon adenocarcinoma model (168). Additionally, cGAMP can enhance the efficacy of the chemotherapy drug 5-FU while reducing its toxic side effects, demonstrating its potential as a novel immunotherapy drug in cancer treatment (168). Notably, the proportion of non-nucleotide small-molecule agonists has been increasing in recent years, as they offer advantages such as high oral bioavailability (161), long half-life (169), strong target specificity (147), and low production costs (147). For example, the non-nucleotide small-molecule agonist MSA-2 demonstrates significant tumor suppression and sustained immunotherapeutic effects when administered orally or via subcutaneous injection, and can selectively activate STING in tumors while reducing systemic toxicity. When combined with PD-1 ICIs, it improves effectiveness while maintaining good tolerability (161). Additionally, cancer vaccines based on STING agonists hold great application potential. For instance, STINGVAX effectively activates the cGAS-STING signaling pathway, stimulating innate and adaptive anti-tumor immune responses, and when combined with PD-1 blockers, it significantly enhances efficacy (170).

**Table 4:** cGAS or STING agonists in anti-tumor immunity.

Target	Type	Name	Mechanism	Effect	Reference
STING	Cyclic Dinucleotides (CDNs)	ADU-S100	Directly activates STING, inducing potent IFN-I and T cell infiltration	Promotes IFN-I production, enhancing anti-tumor immune responses	(157)
		MK-1454	High-affinity binding to STING, inducing IFN-β and pro-inflammatory cytokines	Significantly induces cytokine secretion, exerting anti-tumor effects	(158)
		E7766	Cross-ring bridge linking U-shaped active conformation, improving STING activation efficiency	Broadly activates human STING variants, inducing long-term immune memory, and enhancing anti-tumor effects	(159)
	Non-nucleotide class	SR-717	The structure mimics cGAMP and competitively binds to STING	Enhances T cell killing activity and anti-tumor immune response	(160)
		MSA-2	It forms dimers to activate STING, enhancing stability	Precisely activates STING, enhancing the safety and efficacy of systemic administration	(161)
		di-ABZI	High binding affinity and selectivity activate STING via an open conformation	Efficiently induces IFN-β, enhancing antiviral and anti-tumor immunity	(162)
	Nano-delivery systems	cGAMP-NPs	Liposomal nanoparticles efficiently deliver cGAMP, protecting its stability	Promotes the formation of an anti-tumor microenvironment	(163, 164)
cGAS	Metal-based agonists	Mn²^+^ and its complexes	Enhances DNA damage and directly activates the cGAS-STING pathway	Stimulates tumor-specific T cells to kill tumor cells, thereby enhancing immune function and anti-tumor activity	(165)
	Protein-based	β-arrestin2	Promotes cGAS recognition of dsDNA through deacetylation, accelerates cGAMP synthesis, and enhances activation of the cGAS-STING pathway	Enhances innate immune reactions and improves clearance of viral infections or tumor cells	(166)

cGAS, cyclic GMP-AMP synthase; STING, stimulator of interferon genes; cGAMP, 2'3'-cyclic GMP-AMP, DNA, deoxyribonucleic acid; CDN, cyclic dinucleotide; IFN-β, interferon-beta; IFN-I, Type I Interferons; NPs, nanoparticles; dsDNA, double-stranded DNA.

In summary, given the crucial role of the cGAS-STING pathway in IBD and CRC, targeting and regulating the cGAS-STING axis has emerged as a potential therapeutic strategy for IBD and CRC. Combining this approach with radiotherapy, chemotherapy, CAR-T therapy, and other modalities can further enhance treatment efficacy. Currently, various inhibitors and agonists targeting the cGAS-STING axis have been created and are gradually being validated in preclinical studies or clinical trials. Several drugs targeting the cGAS-STING pathway have shown promise in treating IBD and CRC, as shown in **Table 5**. Although the cGAS-STING route shows great potential in anti-infection, inflammation regulation, and tumor immunity, there are still many shortcomings and challenges in terms of precise delivery, safety, and efficacy. Addressing these issues will not only promote the clinical translation of cGAS-STING pathway-targeted therapy but also provide insights for other immune regulation strategies.

**Table 5:** Drugs targeting the cGAS-STING pathway for the treatment of IBD and CRC.

Name	Target	Mechanism	Effect	Delivery Strategy	Reference
H-151	STING	Targets the CDN-binding site of STING, inhibiting its activation	Shows anti-inflammatory effects in colitis models	Oral lipid nanocapsules	(138)
RU.521	cGAS	Inhibits cGAS, reducing cGAMP levels and phosphorylation of STING, TBK1, and IRF3 in the colon	Demonstrates alleviating effects in colitis models of GSDMD-deficient mice	Intraperitoneal injection	(137)
Compound 3	cGAS	Covalently binds to Cys419 of cGAS, inhibiting substrate binding	Reduces inflammation in DSS-induced colitis mouse models	Intraperitoneal injection	(171)
Compound Sophora Decoction	cGAS	Regulates macrophage polarization and suppresses inflammation by inhibiting cGAS	Alleviates inflammation in UC models	Oral gavage	(172)
Quercetin	cGAS-STING	Inhibits the cGAS-STING pathway to modulate macrophage polarization	Exerts therapeutic effects in DSS-induced UC	Intraperitoneal injection	(173)
DisitamabVedotin	STING	Inhibits HER2-STING binding, relatively activating the cGAS-STING pathway	Suppresses tumor growth in CRC models and enhances sensitivity to immunotherapy	Intravenous injection	(146)
HY041004	cGAS	Enhances cGAS enzymatic activity and inhibits M2 macrophage polarization	Reprograms the anti-tumor immune microenvironment, exerting anti-CRC effects	Oral gavage	(174)
Andrographolide	cGAS-STING	Targets VDAC, activating the cGAS-STING-IFN-β axis	Inhibits tumor growth and remodels the TME in CRC	Intraperitoneal injection	(175)
MTX+TP5	cGAS-STING	MTX induces DNA damage to activate cGAS-STING; TP5 promotes T-cell activation	Demonstrates significant anti-tumor immune activity and tumor suppression	Tail vein injection	(176)

cGAS, cyclic GMP-AMP synthase; STING, stimulator of interferon genes; CDN, cyclic dinucleotide; cGAMP, 2'3'-cyclic GMP-AMP; TBK1, TANK-binding kinase 1; IRF3, interferon regulatory factor 3;GSDMD, gasdermin D;UC, ulcerative colitis; DSS, dextran sulfate sodium;HER2, Human epidermal growth factor receptor 2; CRC,colorectal cancer;VDAC, voltage-dependent anion channel; TME, tumor microenvironment; IFN-β, interferon-beta; DNA, deoxyribonucleic acid;

## 7 Challenges and limitations

Despite significant progress in elucidating the role of the cGAS-STING pathway in IBD and CRC, several critical scientific questions and challenges remain unresolved in this field. For instance, while most studies have focused on immune cell components—such as macrophages, dendritic cells, natural killer cells, and T cells, the impact of non-immune cells (e.g., epithelial and stromal cells)and their functional contributions to intestinal diseases remains insufficiently supported by experimental evidence (9, 173). Expanding investigations to non-immune cell populations may help uncover intricate cell-cell interactions and provide novel insights for developing more cell-targeted therapeutic strategies and interventions. Additionally, preclinical research predominantly relies on genetically engineered mouse models, which exhibit substantial differences from humans in terms of intestinal anatomy, immune cell repertoire, and microbiome composition (177). Given the structural and regulatory divergence of cGAS-STING pathway components across species, more rigorous evaluation of preclinical drug candidates is warranted to ensure their translatability and applicability in clinical trials (178). Also, in the context of therapeutic applications, existing STING agonists/antagonists often suffer from poor tissue specificity and high systemic toxicity, with severe adverse events, including cytokine release syndrome and autoimmune-like reactions, previously reported in clinical trials (179). Moreover, both IBD and CRC exhibit considerable disease heterogeneity, with significant variations in cGAS-STING pathway activity across different molecular subtypes within the TME. However, reliable biomarkers for clinical subtyping and personalized treatment remain lacking. From a technical standpoint, the low abundance of pathway components in human tissue samples and the substantial interindividual variation in gut microbes further complicate the challenges of sensitivity in detection and mechanistic studies (180). Addressing these fundamental and translational challenges is a critical prerequisite for translating basic research on the cGAS-STING pathway into clinically viable therapies.

## 8 Conclusion and future perspectives

Recently, the cGAS-STING signaling pathway has garnered significant attention for its crucial role in regulating the innate immune response and is emerging as a promising therapeutic target for intestinal diseases. cGAS plays a critical role in host defense, inflammatory responses, and tumor immune regulation by recognizing exogenous and endogenous DNA. Studies have shown that extracellular vesicles (EVs) derived from microorganisms and tumors can activate the cGAS-STING pathway by delivering their specific DNA. Besides mediating the classic IFN-1 response, this pathway also regulates various biological processes, including autophagy, ER stress, and senescent cell death. The cGAS-STING signaling pathway has a "double-edged sword" effect in the intestinal environment. On one hand, normal activation levels of cGAS-STING help maintain homeostasis of the intestinal barrier and promote tumor-suppressing immune responses. On the other hand, extended periods of unusual activation can lead to excessive inflammation, resulting in damage to the intestinal epithelium and an imbalance in the immune microenvironment. As research into the cGAS-STING signaling pathway advances, the clinical development of targeted agents has gained significant attention. STING agonists can enhance the anti-tumor effects of CRC treatment, while pathway inhibitors may improve the clinical prognosis of IBD and certain colorectal carcinomas. We hope that in the future, more biomarker-based targeted regulatory strategies can be developed to advance their precise clinical application in IBD and CRC.

## Glossary

cGAS: Cyclic GMP-AMP synthase
STING: Stimulator of interferon genes
dsDNA: Double-stranded DNA
cGAMP: 2'3'-cyclic GMP-AMP
ATP: Adenosine triphosphate
GTP: Guanosine 5'-triphosphate
NF-κB: Nuclear factor-kappaB
IRF3: Interferon regulatory factor 3
CRC: Colorectal cancer
IBD: Inflammatory bowel disease
NTase: C-terminal nucleotidyltransferase
ER: Endoplasmic reticulum
ERGIC: ER-Golgi intermediate compartment
TBK1: Threonine protein kinase 1
IκBα: Inhibitor kappa B alpha
TNF: Tumor necrosis factor
TREX1: Three-prime repair exonuclease 1
COPII: Coat protein complex II
DNaseII: Deoxyribonuclease II
SAMHD1: SAM domain and HD domain-containing protein 1
AIM2: Absent in melanoma 2
AKT: Protein kinase B
FI16: Interferon-inducible protein 16
TRIM41: Tripartite motif-containing protein 41
ENPP1: Ectonucleotide pyrophosphatase/phosphodiesterase 1
AMFR: Autocrine motility factor receptor
MUL1: Mitochondrial E3 ubiquitin ligase 1
USP13: Ubiquitin-specific peptidase 13
ULK1: Unc-51 like autophagy activating kinase 1
UPR: Unfolded protein response
ATM: Ataxia telangiectasia mutated
ATR: Ataxia telangiectasia and Rad3-related
CD: Crohn's disease
UC: ulcerative colitis
sIgA: Secretory immunoglobulin A
MUC1: Mucins 1
ILC1: Innate lymphoid cells 1
IFN-I: Interferon I
ISGs: Interferon-stimulated genes
LP: Lamina propria
mtDNA: Mitochondrial DNA
DSS: Dextran sulfate sodium
CDNs: Cyclic dinucleotides
ncDNA: Nuclear DNA
mEVs: Microvesicles
LDCP: Lysosome-dependent pyroptosis
LMP: Lysosomal membrane permeabilization
GSH: Glutathione
CMV: Cytomegalovirus
APC: Antigen-presenting cell
DC: Dendritic cell
ROS: Reactive oxygen species
CIN: Chromosomal instability
SASP: Senescence-associated secretory phenotype
SLC19A1: Solute carrier family 19 member 1
CCR7: C-C chemokine receptor type 7
TME: Tumor microenvironment
NK: Natural killer
dMMR: Defective mismatch repair
MSI: Microsatellite instability
pMMR: Proficient DNA mismatch repair
MSS: Microsatellite stability
ICI: Immune checkpoint inhibitor
GSDMD: Gasdermin D
GLP-2: Glucagon-like peptide-2
Daxx: Death-associated protein
ICD: Immunogenic cell death
PREX2: Phosphatidylinositol-3,4,5-trisphosphate RAC exchanger 2
PKcs: Catalytic subunit
PRMT6: Protein arginine methyltransferase 6
PD-1: Programmed cell death protein 1
SHP1: SH2 domain-containing phosphatase 1
GSDME: Gasdermin E
mtROS: Mitochondrial reactive oxygen species
RT: Radiation therapy
pHER2: Phosphorylated HER2
NO2-FAs: Nitro-fatty acids
Cys: Cysteine
